# Intravenous immunoglobulins improve skin fibrosis in experimental models of systemic sclerosis

**DOI:** 10.1038/s41598-023-42464-9

**Published:** 2023-09-12

**Authors:** Silvia Speca, Meryem-Maud Farhat, Manel Jendoubi, Thomas Guerrier, Sébastien Sanges, Delphine Staumont-Sallé, Eric Hachulla, Sylvain Dubucquoi, Vincent Sobanski, Aurore Collet, David Launay

**Affiliations:** 1 University of Lille, Inserm, CHU Lille, U1286-INFINITE—Institute for Translational Research in Inflammation, Lille, France; 2 Département de Médecine Interne Et Immunologie Clinique, Centre de Référence Des Maladies Auto-Immunes Systémiques Rares du Nord Et Nord-Ouest de France (CeRAINO), CHU Lille, Lille, France; 3 Service de Dermatologie, CHU Lille, Lille, France; 4 Institut d’Immunologie, Centre de Biologie Pathologie, CHU Lille, Lille, France

**Keywords:** Systemic sclerosis, Chronic inflammation

## Abstract

Systemic sclerosis (SSc) is the most severe systemic autoimmune disease with currently no cure. Intravenous immunoglobulins (IVIg) are an attractive candidate in this disease to counteract inflammation and fibrosis but data are scarce and conflicting. This study, assessed the effects of IVIg in a murine HOCl-induced model of SSc. We showed that IVIg prevented skin inflammation and fibrosis, by mitigating the immune cell infiltration (*p* = 0.04), proinflammatory cytokines gene overexpression (IL1β, *p* = 0.04; TNFα, *p* = 0.04; IL6, *p* = 0.05), skin and dermal thickening (*p* = 0.003 at d21 and *p* = 0.0003 at d42), the expression markers of fibrosis, such as αSMA (*p* = 0.031 for mRNA and *p* = 0.05 for protein), collagen (*p* = 0.05 for mRNA and *p* = 0.04 for protein, *p* = 0.05 for the hydroxyproline content) and fibronectin (*p* = 0.033 for mRNA). Moreover, IVIg prevented HOCl-induced alterations in splenic cell homeostasis. When administered in curative mode, despite their ability to reduce skin and dermal thickness (*p* < 0.0001 and *p* = 0.0002), IVIg showed partial or more mixed effects on skin inflammation and established fibrosis. These data favor further clinical trials in SSc patients on the potential efficiency of early and/or repeated IVIg administration.

## Introduction

Systemic sclerosis (SSc) is a severe chronic multi-system inflammatory disease, whose pathophysiology combines vasculopathy, abnormal adaptive and innate immune system activation and aberrant fibrogenesis^1,2^. Consequently, available treatments in SSc include vasoactive molecules, immunosuppressants and antifibrotic drugs. Yet, there is still no cure and the response to treatment is modest. Moreover, recent trials assessing biologics have been negative, reinforcing the need to enrich the armamentarium for this disease^3^.

Intravenous immunoglobulins (IVIg) are human polyspecific IgG used in various immune-mediated inflammatory diseases^4^. Several arguments support the possible beneficial effect of IVIg in SSc, although the effects of IVIg in SSc are still elusive due to the paucity of data. First, some mechanisms of action of IVIg could interfere with SSc pathophysiology, by modulating the proliferation and differentiation of B cells and their antibody production and controlling pro-inflammatory cytokine release^5,6^. Second, preclinical data suggest a possible efficacy of IVIg in experimental models of SSc, although studies are scarce and only assessed the preventive effect of IVIg, i.e. the ability of IVIg to prevent the development of the model when started on the first day of model induction. Blank et al. (2002) showed that preventive IVIg reduced the amount of skin collagen and inhibited the secretion of transforming growth factor (TGF)-β1 and interleukin (IL)-4 by splenocytes, in tight-skin mice^7^. Kajii et al. (2011) showed that preventive IVIg decreased the excessive collagen accumulation in the bleomycin model as well as decreased the expression of pro-inflammatory and pro-fibrotic cytokines or chemokines like TGF β1 and the monocyte chemoattractant protein-1^8^. Third, clinical data suggest a possible efficacy of IVIg in SSc. The only available randomized-controlled study used a single course of IVIg vs placebo in SSc^9^. The primary endpoint, i.e. the change in the modified Rodnan skin score (mRSS) at 12 weeks, was negative. However, a second injection was associated with a significant decrease in mRSS^9^. Other uncontrolled case series and literature reviews suggested that IVIg were associated with: (i) a decrease in mRSS in SSc^10–12^; (ii) an increase in IFNγ in the skin of SSc patients; (iii) a corticosteroid-sparing benefit in SSc-associated myopathy, without any effect on the skin in this disease^13^. In our nationwide clinical study, we also found that IVIg administration was associated with a better muscle outcome in SSc patients but only stability of skin and lung involvement^14^.

**Figure 1:**
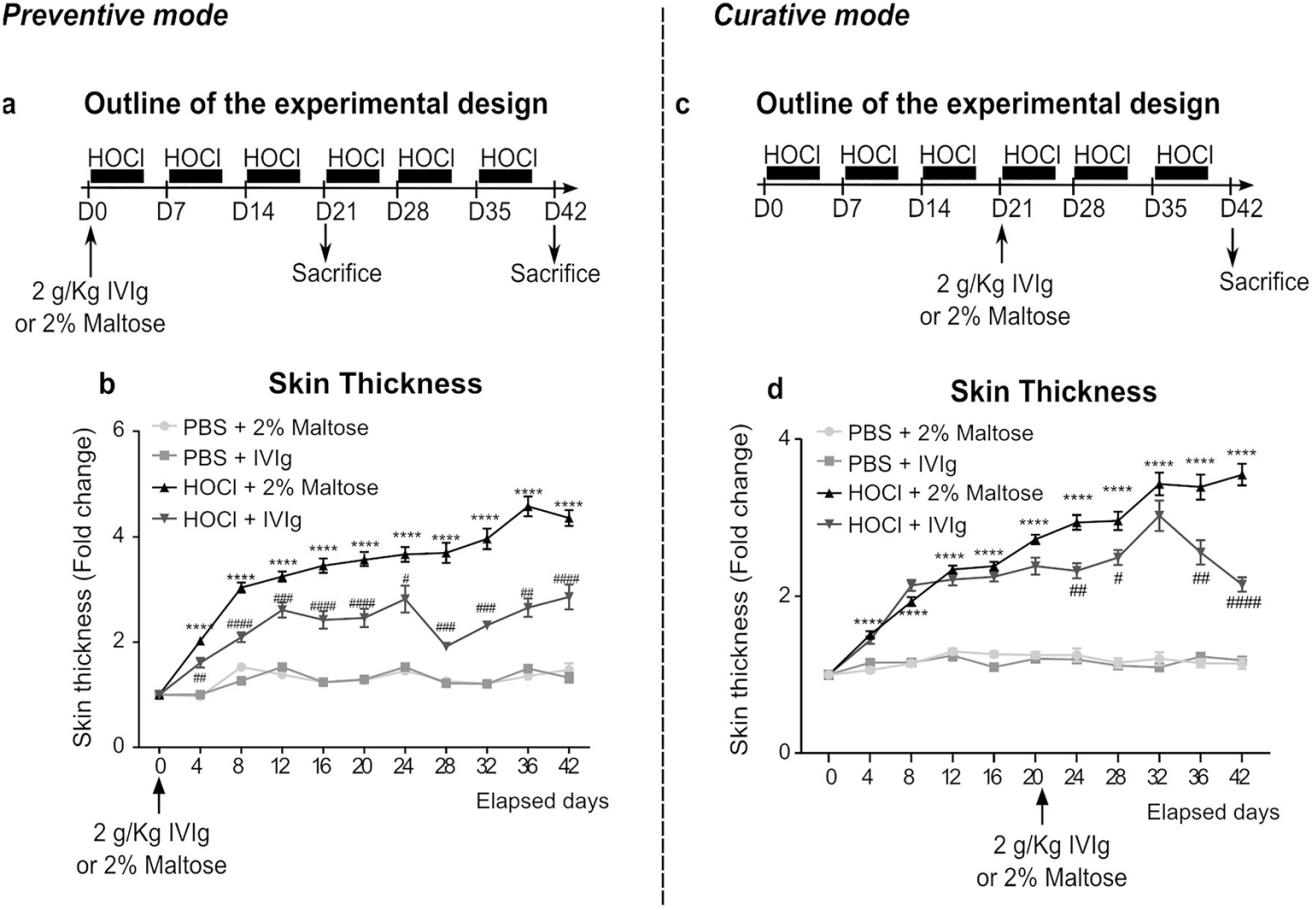
Study design and clinical features following the IVIg administration with preventive and curative intent. Outlines of the experimental design for (**a**) preventive and (**c**) curative protocols. Mice were randomized to: PBS + 2% Maltose (n = 50), PBS + IVIg (n = 50), HOCl + Maltose (n = 50), HOCl + IVIg (n = 50) groups. Each group included 30 mice for the preventive protocol and 20 mice for the curative protocol. 300 µL of HOCl or PBS were administered by daily intradermal injection into the shaved backs of mice until sacrifice. 2 g/Kg of IVIg were administered in a single retro-orbital injection at d0 with preventive intent, and at d21 with curative intent. (**b**,**d**) Curves depicting skin thickness variation compared to D0, measured every 4 days during the whole experimental protocols, for each group. Data are quoted as mean ± SEM; *p < 0.05 versus PBS + 2% Maltose; **p < 0.01 versus PBS + 2% Maltose; ***p < 0.001 versus PBS + 2% Maltose; ****p < 0.0001 versus PBS + 2% Maltose; ^##^*p* < 0.01 vs HOCl + 2% Maltose; ^###^*p* < 0.001 versus HOCl + 2% Maltose. Data were analyzed by a Two-way ANOVA test with Tukey’s multiple comparisons.

Altogether, these data suggest that IVIg might be interesting in SSc but results are conflicting, most often due to uncontrolled studies without a placebo, in a limited number of patients, who possibly already received immunosuppressants. Preclinical data are scarce, and mechanisms of action are not fully understood. To fill this gap, the present study aimed to investigate whether IVIg administration was able to prevent and improve skin fibrosis in experimental models of SSc and to characterize their effects. For this aim, we used the previously described hypochlorous acid (HOCl)-induced model of SSc (Servettaz et al., 2009). IVIg were administered by a single retro-orbital injection at d0 (with preventive design) or d21 (with curative design) of HOCl administration (Fig. 1a and c). Results were confirmed on a second validated experimental murine model of SSc induced by the bleomycin (BLM) administration (Supplementary Figure S1a).

## Results

### Intravenous immunoglobulins both prevent and reduce skin thickness in HOCl-receiving mice

To monitor the development and severity of the skin fibrosis induced by the HOCl administration and the beneficial effect of IVIg treatment, we first focused on the cutaneous and dermal layer thickness, as well as the histological evaluation of skin architecture. HOCl mice receiving vehicle, showed a gradual significant increase in skin thickness, starting from d4 and reaching a peak of a fourfold increase compared to PBS (*p* < 0.001) at the end of the disease induction protocol (Fig. 1b,d). Both preventive and curative IVIg administration was able to antagonize the HOCl-induced skin thickening, with a rapid and progressive effect observed as soon as the 4th day after the start of IVIG administration.

**Figure 2:**
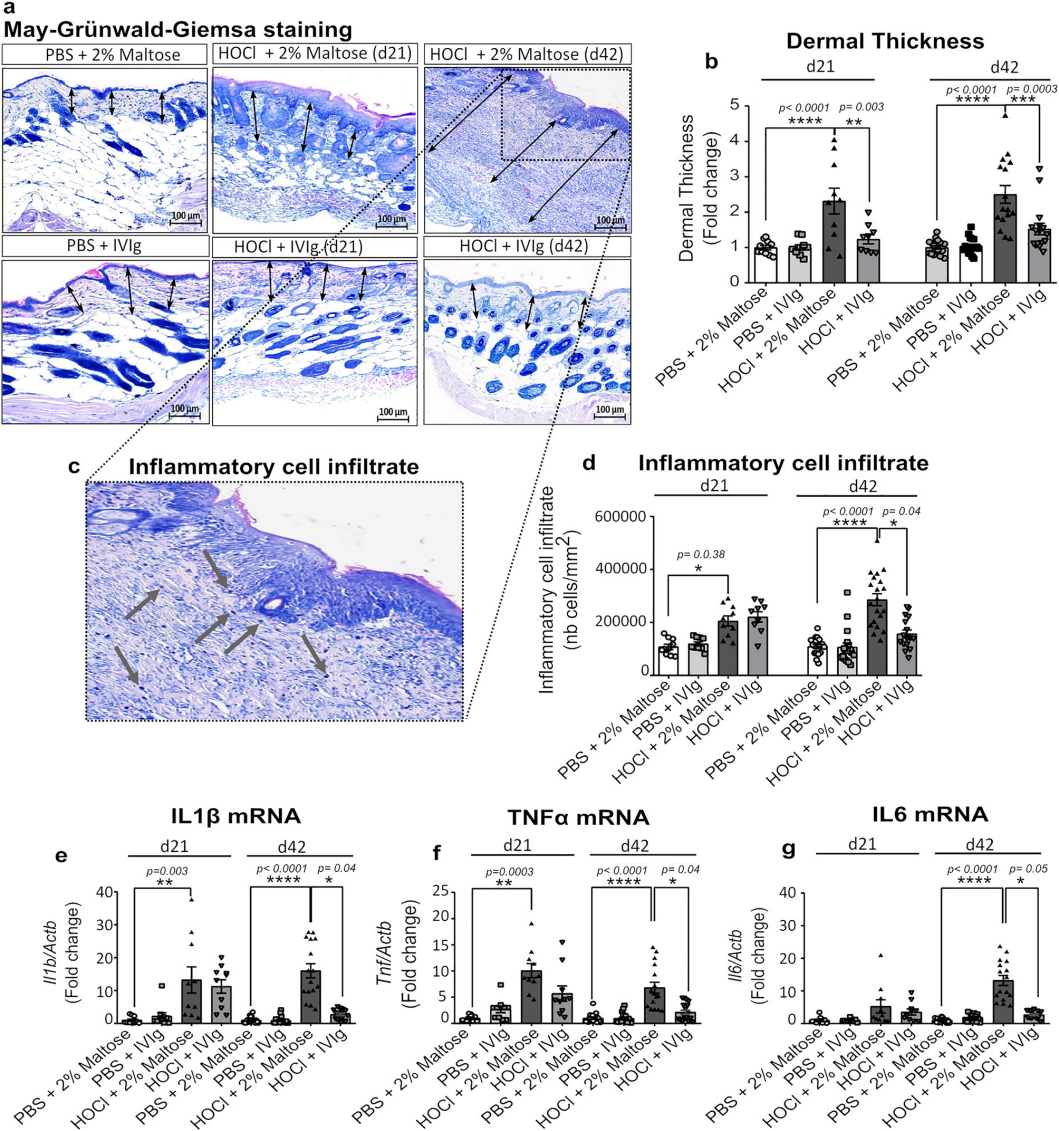
Histological skin evaluation and inflammatory response of all mice included in the preventive design. (**a**) Representative microphotographs of MGG stained skin sections scanned at 20X magnification. (**b**) Histograms depicting Image J software-assisted measures of dermal thickness. (**c**) Enlargement of a representative microphotograph where inflammatory cells are visualized as blue spots (see gray arrows). (**d**) Histograms of Image J software-assisted counting of cellular infiltrates in MGG stained skin sections. mRNA expression levels of pro-inflammatory cytokines, such as (**e**) *Il1b*, (**f**) *Tnf* and (**g**) *Il6*, evaluated by qRT-PCR, in frozen skin tissues. Data represent the fold change compared to PBS + 2% Maltose group and are quoted as mean ± SEM and analyzed by Kruskal–Wallis test with Dunn’s multiple comparisons; *p < 0.05; **p < 0.01; ***p < 0.001; ****p < 0.0001.

**Figure 3:**
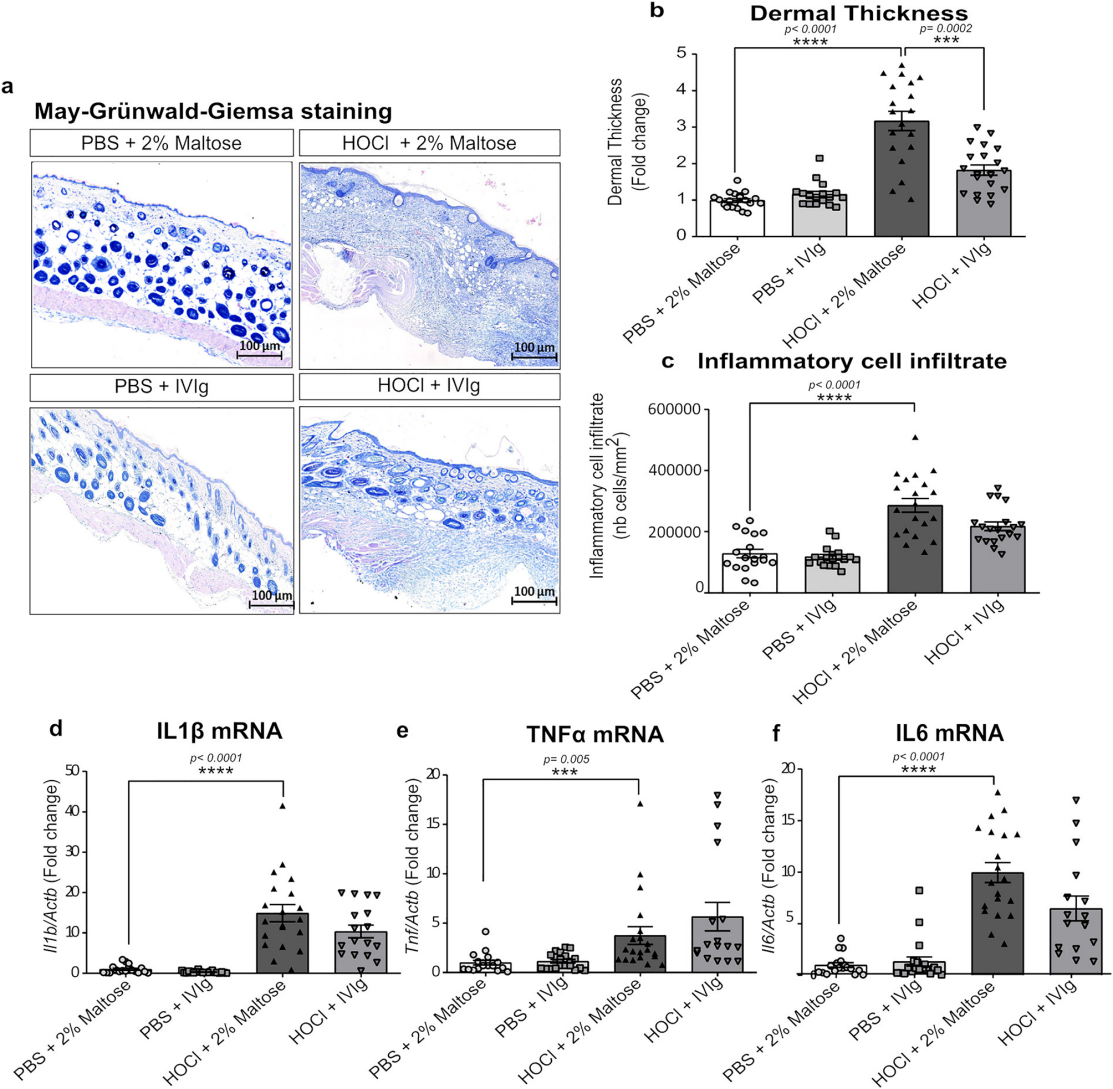
Histological skin evaluation and inflammatory response of all included mice in the curative design. (**a**) Representative microphotographs of MGG stained skin sections scanned at 20X magnification. (**b**) Histograms depicting Image J software-assisted measures of dermal thickness. (**c**) Histograms for Image J software-assisted counting of cellular infiltrates in MGG stained skin sections. mRNA expression levels of pro-inflammatory cytokines, such as (**d**) *Il1b*, (**e**) *Tnf* and (**f**) *Il6*, evaluated by qRT-PCR, in frozen skin. Data represent the fold change compared to PBS + 2% Maltose group and are quoted as mean ± SEM and analyzed by Kruskal–Wallis test with Dunn’s multiple comparisons; *p < 0.05; **p < 0.01; ***p < 0.001; ****p < 0.0001.

Histological measurements on digitalized images of whole May Grünwald-Giemsa (MGG)-stained skin sections, showed that the HOCl-induced skin thickening was essentially explained by a significant increase in the dermal layer, by 2.3 ± 0. 0.36 folds (*p* < 0.0001) at d21 and 2.5 ± 0.16 folds (*p* < 0.0001) (in the preventive experimental setting) or 3.17 ± 0.26 folds (*p* < 0.0001) (in the curative experimental setting) at d42, compared to the control group (Fig. 2a). Histological observations showed an evident alteration of skin morphology, with a loss of skin integrity and hypodermal layer in the HOCl group (Figs. 2a, 3a). Beneficial effects on the altered cutaneous architecture and the dermal thickening were observed when IVIg were administered both in preventive (at d21, *p* < 0.003 and d42, *p* < 0.0003) (Fig. 2b) and curative (*p* < 0.002) designs (Fig. 3b).

### Intravenous immunoglobulins prevent but do not reverse established skin inflammation

To evaluate the anti-inflammatory potential of IVIg administration in HOCL-receiving mice, we evaluated the presence of inflammatory cell infiltrates on digitalized images of whole May Grünwald-Giemsa (MGG)-stained histological skin sections, as well as the gene expression levels of pro-inflammatory cytokines. Mice treated with HOCl showed evident skin hyperplasia which was associated with a greater density of inflammatory cells in dermal and hypodermal layers (the grey arrows in Fig. 2c), resulting in a significant increase in the number of cell/mm^2^ at both d21 (*p* = 0.038) and d42 (*p* < 0.0001) compared to control mice (Figs. 2c,d and 3c). The HOCl administration caused a significant increase in gene expression levels of the main inflammatory cytokines in skin specimens of HOCl-receiving mice, such as interleukin (IL)-1β and tumor necrosis factor (TNF)-α already at d21 by at least 6 to 10 folds (*p* = 0.003 and *p* = 0.0003, respectively). The significant upregulation of these cytokines was maintained throughout the study (*p* < 0.0001). The later phase of the HOCl-induced disease was also associated with significantly increased levels of IL-6 by 13.2 ± 1.53 folds, compared to the control group (*p* < 0.0001).

The preventive administration of IVIg was associated with a lower density of cutaneous inflammatory cells infiltrate at d42 (*p* = 0.04) (Fig. 2d), together with a normalization of the skin gene expression of inflammatory cytokines (Fig. 2e,f,g) when compared to vehicle. Conversely, in the curative experimental design, IVIg did not induce any significant effect on the established inflammatory infiltrate and skin gene expression levels of main inflammatory cytokines IL-1β, TNF-α and IL-6 (Fig. 3c,d,e,f).

### Intravenous immunoglobulins reduce the overexpression of fibrosis-associated genes and the skin collagen content in HOCl-receiving mice, with more mitigated effects in established fibrosis

**Figure 4:**
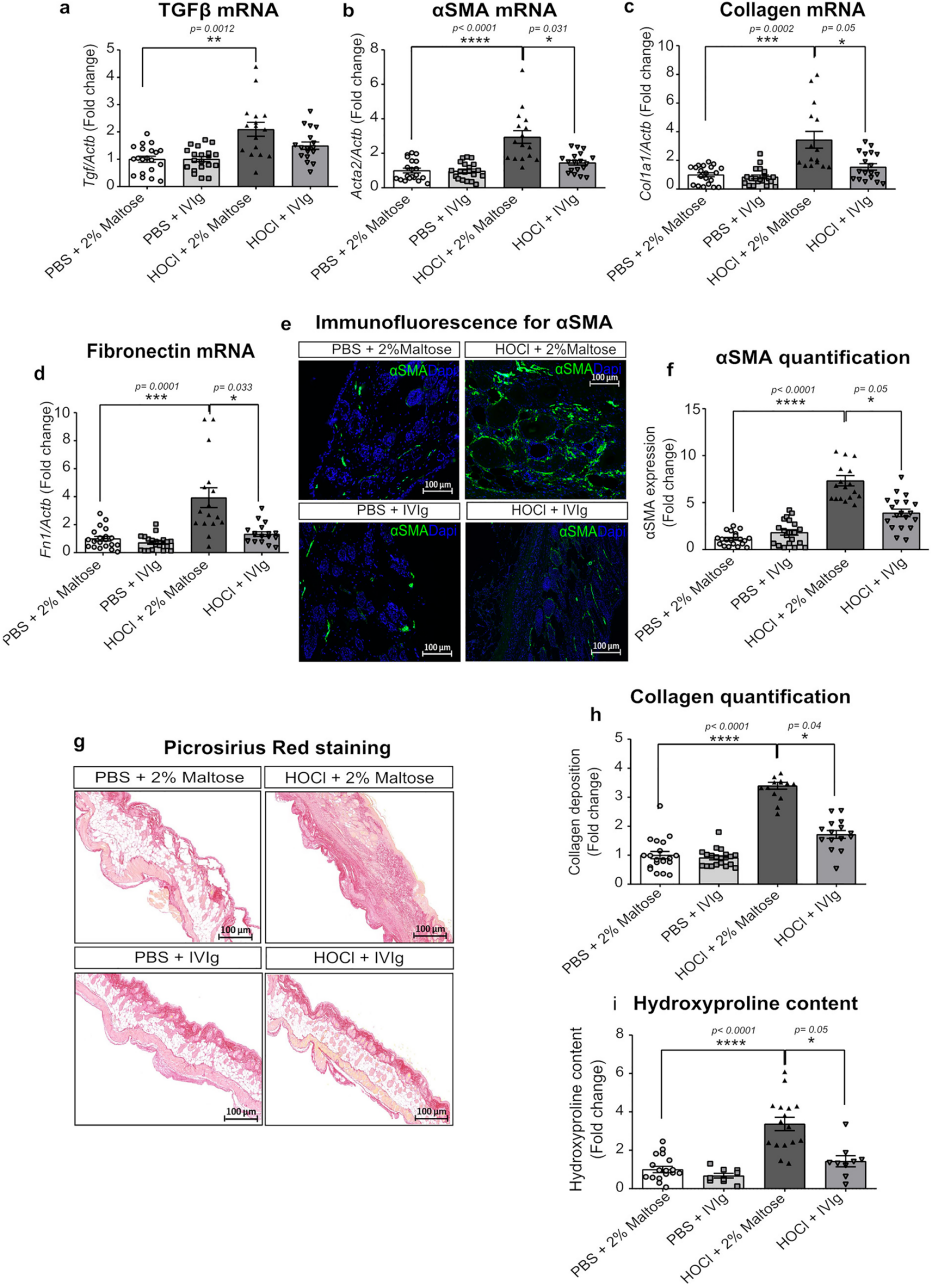
Pro-fibrotic markers of all included mice in the preventive intent. (**a**) Histograms depicting mRNA expression levels of (**a**) *Tgfb*, (**b**) *Acta2*, (**c**) *Col1A1* and (**d**) *Fn1* genes quantified on frozen skin samples by RT-PCR. (**e**) Representative microphotographs on skin sections stained by immunofluorescence for αSMA (green) scanned at 40X magnification. Nuclei were counterstained using DAPI (bleu). (**f**) Representative microphotographs for Picrosirius Red staining of skin sections for collagen fibers (red) and scanned at 20X magnification. (**g**) Histograms for αSMA and (**h**) collagen deposition quantified on three microscope fields, using the threshold detection method. (**i**) Histogram depicting hydroxyproline content levels. Data represent the fold change compared to PBS + 2% Maltose group and are quoted as mean ± SEM and analyzed by Kruskal–Wallis test with Dunn’s multiple comparison; *p < 0.05; **p < 0.01; ***p < 0.001; ****p < 0.0001.

To confirm the potential efficiency of IVIg administration in improving HOCl-induced skin fibrosis, we also evaluated their role in controlling the production of TGF-β (coded by the gene *Tgfb* gene), a pivotal pro-fibrotic growth factor, the expression of αSMA (coded by the gene *Acta2* gene), the myofibroblasts activation marker and the production of extracellular matrix components, such as collagen and fibronectin. As expected, we showed a significant upregulation of mRNA expression levels for *Tgfb* (*p* = 0.0012), *Acta2* (by 2.94 ± 0.37 folds, *p* < 0.0001), as well as for genes coding for collagen, *Col1a1 (*by 3.43 ± 0.58 folds*, p* = 0.0002 and fibronectin, *Fn1* (3.92 ± 0.7 folds, *p* = 0.0001) in the HOCl group at d42 (Fig. 4a,b,c,d). When preventively administered, IVIg downregulated the HOCl-induced overexpression of the pro-fibrotic genes with a significant reduction of mRNA expression levels by 51% for *Acta2* (*p* = 0.031), 55% for *Col1a1* (*p* = 0.049) and 66% for *Fn1* (*p* = 0.033), compared to HOCl mice treated by vehicle (Fig. 4b,c,d). *Tgfb* gene expression levels tended to be reduced (*p* = 0.075) by preventive IVIg infusion (Fig. 4a). To evaluate the IVIg effects at the protein levels, we also quantified the number of αSMA-positive cells by immunofluorescence and collagen production by picrosirius red staining (PRS). Daily HOCl administration caused a significant increase in the number of αSMA-positive cells (by 7.33 ± 0.55 folds, *p* < 0.0001), as well as a higher collagen fibers deposition (3.4 ± 0.12 folds, *p* < 0.0001) and hydroxyproline content (3.4 ± 0.35 folds, *p* < 0.0001), compared to control mice (Fig. 4e,f,g,h,i). Preventive IVIg administration was able to reduce the HOCl-associated number of α-SMA-positive cells by 47% (*p* = 0.05), collagen synthesis by 49% (*p* = 0.044) and the hydroxyproline content by 58% (*p* = 0.048) (Fig. 4i).

**Figure 5:**
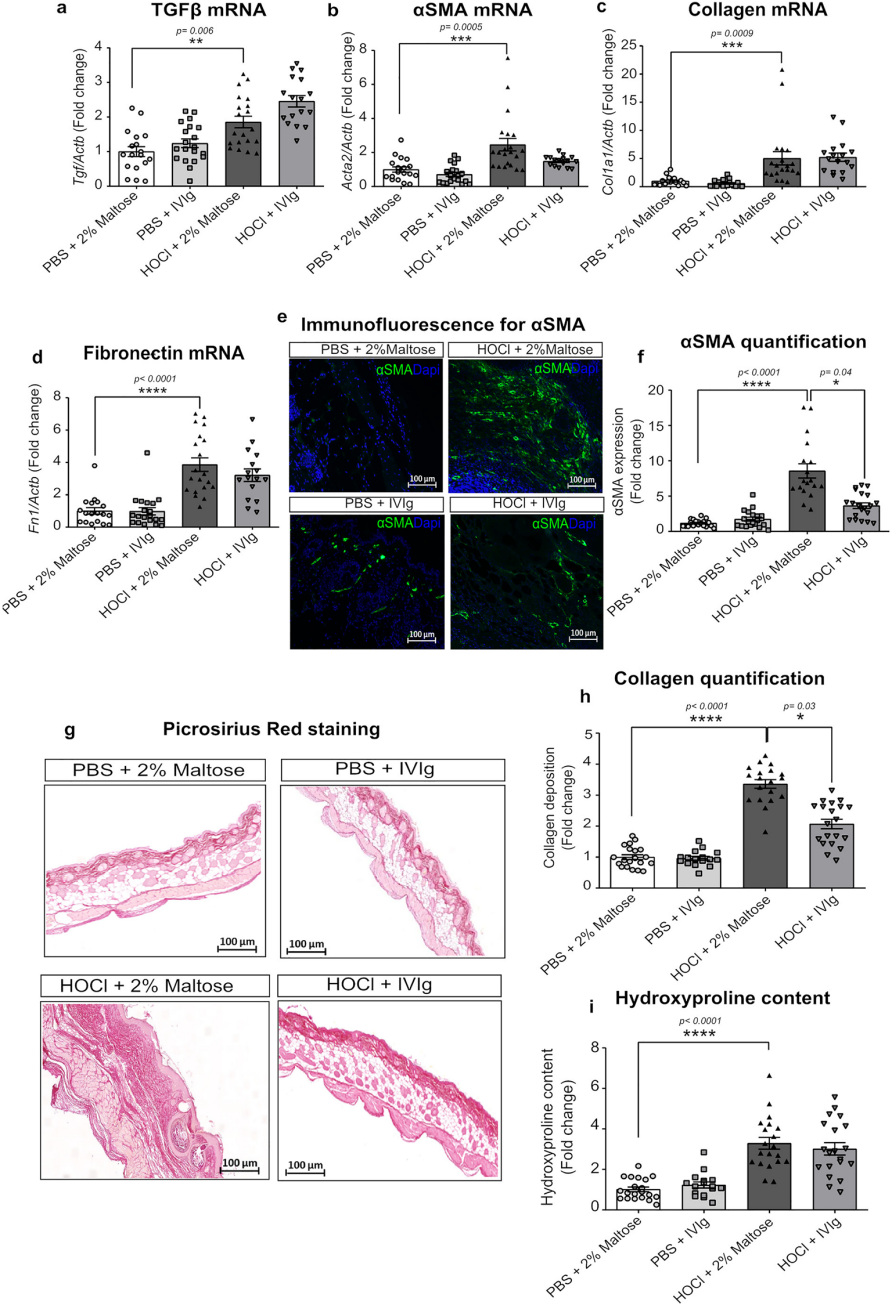
Pro-fibrotic markers of all included mice in the curative design. (**a**) Histograms depicting mRNA expression levels of (**a**) *Tgfb*, (**b**) *Acta2*, (**c**) *Col1a1* and (**d**) *Fn1* genes quantified on frozen skin samples by qRT-PCR. (**e**) Representative microphotographs on skin sections stained by immunofluorescence for αSMA (green) scanned at 40X magnification. Nuclei were counterstained using DAPI (bleu). (**f**) Representative microphotographs for Picrosirius Red staining of skin sections for collagen fibers (red) scanned at 20X magnification. Digital images were processed with Zeiss LSM Browser. (**g**) Histograms for αSMA and (**h**) collagen deposition quantified on three microscope fields, using the threshold detection method. (**i**) Histogram depicting hydroxyproline content levels. Data represent the fold change compared to PBS + 2% Maltose group and are quoted as mean ± SEM and analyzed by Kruskal–Wallis test with Dunn’s multiple comparisons; *p < 0.05; **p < 0.01; ***p < 0.001; ****p < 0.0001.

Conversely, in the curative experimental setting, gene expression levels of *Tgfb*, *Acta2, Col1A1* and *Fn1* remain unchanged, as well as the amount of αSMA-positive cells in the skin (Fig. 5a,b,c,d,e,f) after IVIg infusion when compared to HOCl-receiving mice at d42. A significant effect of IVIg was only observed for the extent and density of collagen fibers, which was reduced by 40% (p = 0.0323) in HOCl mice (Fig. 5g,h) treated by IVIg versus vehicle.

### Anti-fibrotic effect of preventive IVIg is confirmed in the bleomycin (BLM) model

The BLM-induced model of SSc is the best-known and most used model to date. However, the substantial difference with the HOCl experimental model is represented by the presence of autoantibody-producing cells, which in the BLM model is found only at the level of the gastric mucosa (Ishikawa et al., 2009). Thus, to confirm the preventive anti-fibrotic role of IVIg, we observed their effects in a complementary BLM model. As for the HOCl model, we showed that, when preventively administered, IVIg were able to improve the increase in skin thickness, restore tissue architecture, control dermal thickness and downregulate *Acta2* and *Fn1* gene skin expression, as well as the collagen production, in mice with BLM-associated skin fibrosis (Supplementary Figure S1).

### IVIg prevent but do not reverse HOCl-induced alterations in splenic cells subsets

**Figure 6:**
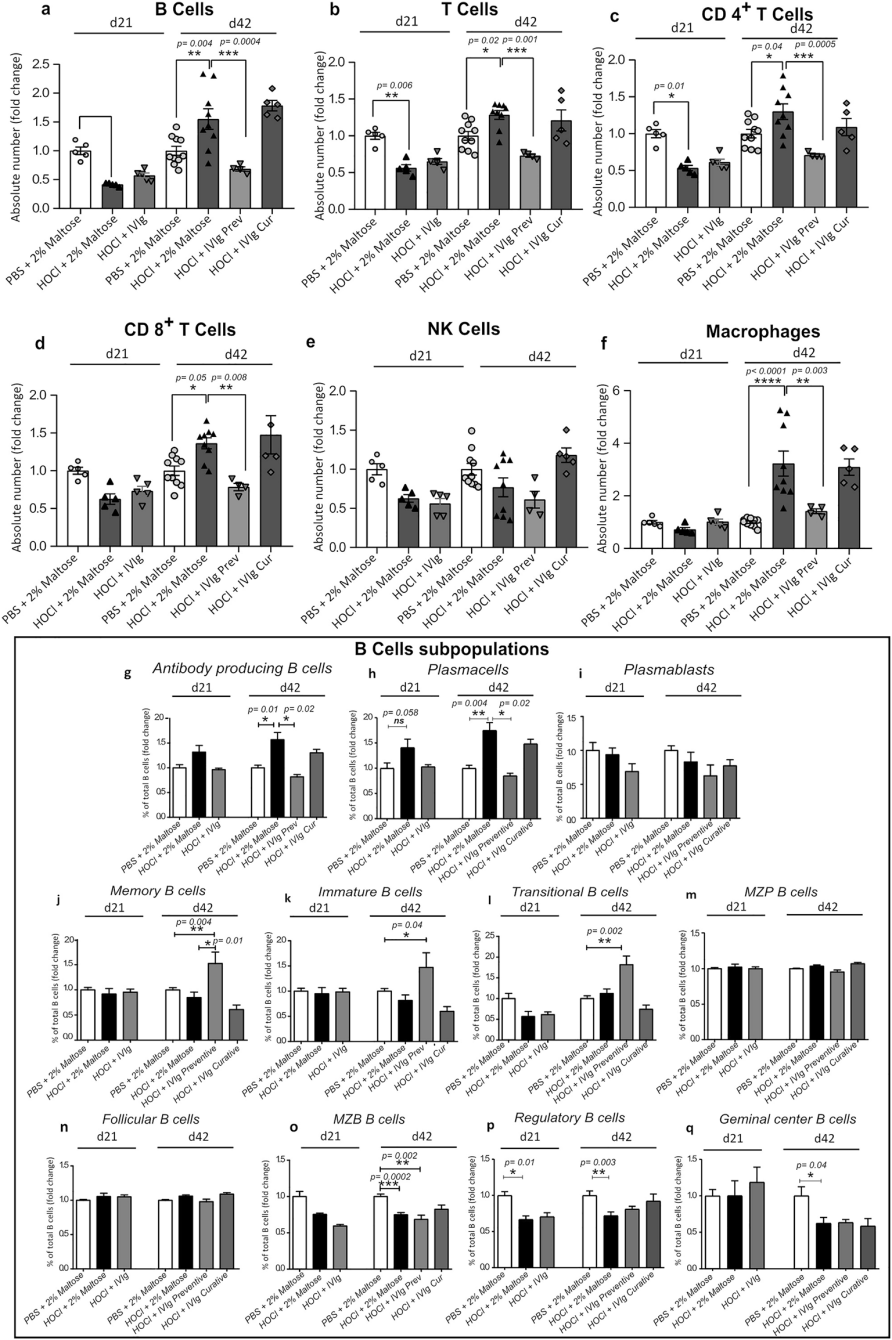
Distribution of the main spleen cell subsets in response to HOCl administration and after IVIg treatment with preventive and curative intents. Histograms depicting the absolute number of (**a**) B cells, (**b**) T cells, (**c**) CD4 + T cells, (**d**) CD8 + T cells, (**e**) Natural Killer (NK) cells, and monocytes–macrophages (**f**). (**g**–**q**) Histograms depicting B cells subpopulations. Data are quoted as the mean of fold change ± SEM versus PBS + 2% Maltose group and analyzed by Kruskal–Wallis test with Dunn’s multiple comparisons; *p < 0.05; **p < 0.01.

As we previously described, the HOCl model is also characterized by alteration in the number and function of immune cell populations (Sanges et al., 2017). In this study, we demonstrated that preventive, but not curative, IVIg administration normalized the increased numbers of splenic CD3^+^ T cells (including CD4^+^ and CD8^+^ T cells) and CD19^+^B cells, as well as CD11b^+^ FSC^hi^ macrophages induced by HOCl injection at d42 when compared to vehicle (Fig. 6a,b,c,d,f). Concerning the natural killer (NK) cells, we did not observe any significant alteration induced by the HOCl administration, nor by preventive or curative IVIg infusion (Fig. 6e). Preventive IVIg administration also normalized the increased numbers of CD138^hi^ CD19^lo/−^ antibody-secreting cells (*p* = 0.004), mainly CD138^hi^ CD19^lo/−^ CD22^−^ plasma cells (*p* = 0.019) at d42 when compared to vehicle (Fig. 6g,h), whereas any significant variation was observed for plasmablasts (Fig. 6i). We also observed that preventive administration of IVIg increased the percentage of immature, transitional and memory B cells at d42 in HOCl mice, when compared to vehicle (Fig. 6j,k,l). Concerning the other B cell subsets, any significant variation was observed when mice received IVIg in the preventive setting (Fig. 6m,n,o,p,q).

## Discussion

This study aimed to demonstrate and characterize the effects of IVIg administration in preventing the development or reversing skin inflammation and fibrosis in experimental models of SSc.

We showed that the preventive use of intravenous immunoglobulins (IVIg) was associated with a significant reduction of skin fibrosis and inflammation, as well as with a normalization of the immune system homeostasis in the HOCl experimental model of SSc. The beneficial effect of preventive IVIg was confirmed in the complementary BLM model. These observations are in line with the 2 existing studies by Blank et al. (2002) and Kajii et al. (2011), showing the ability of IVIg to prevent skin fibrosis and inflammation, as evaluated by histology, transcriptomic analysis of proinflammatory and profibrotic cytokines and collagen content in experimental models of SSc^7,8^. The consistency of results through 3 complementary animal models of SSc, i.e. HOCl, BLM and tight-skin (TSK) mice is a strong argument for the beneficial effect of IVIg in preventing inflammation and fibrosis. Interestingly, Kudo et al. (2013) also showed that IVIg were able to decrease IL-1β serum levels in patients with SSc, yet without significant effects on circulating IL-6 and TNFα^15^. Our study adds a new dimension by assessing the effect of preventive IVIg on immune cell homeostasis. First, we confirm that the HOCl model is associated with an activation of the immune system, as shown by an increased number of B- and T-cells, as well as macrophages. More precisely, this model is associated with an increased number of CD138^hi^ CD19^lo/−^ antibody-producing B cells, including CD138hi CD19^lo/^ CD22^−^ plasma cells. In our study, preventive IVIg were able to rebalance B cell populations, suggesting, first, an immunomodulator effect of IVIg and, second, a possible mechanism of action of IVIg in preventing the development of fibrosis and inflammation. Altogether, our data show that IVIg can prevent the increased expression of pro-inflammatory and pro-fibrotic cytokines, including the transforming growth factor (TGF)-β, normalize alterations of immune cells’ absolute numbers and decrease the number of skin-infiltrated inflammatory cells, which are among the possible mechanisms of action of IVIg that are hypothesized in SSc^4,16^.

To the best of our knowledge, our study is the first to assess the efficacy of IVIg in a curative experimental design i.e., on an established inflammatory and fibrotic pattern. We showed that IVIg can reduce skin thickening and collagen content in this setting. Yet, the effect on inflammation, skin expression of pro-inflammatory and pro-fibrotic cytokines and immune cell homeostasis were non-significant. The explanations are not straightforward, but we can hypothesize that the curative experimental design is more stringent to demonstrate a positive effect on an already established model, after a single injection of IVIg. These mitigated results in the curative experimental design can also shed some light on the discrepancies observed in the literature in SSc patients^13,14^. Altogether, our study also suggests that an early treatment could be more efficient than a late treatment in SSc. It also suggests that one single course of IVIg in established fibrosis could be insufficient, which is in line with the results of the study by Takehara et al^9^.

Our study has some limits. The primary aim of our study was to assess the effect of IVIG on skin involvement. Lung involvement is also an important visceral manifestation of SSc. Yet, in this study, HOCl mice did not develop a sufficient lung fibrosis (data not shown), which precludes any firm conclusion on the effect of IVIG on the lung involvement, which deserves dedicated studiesWe did not assess other possible mechanisms of action of IVIg including their effects on regulatory T cells, as well as on Th17 cells^17,18^.

In conclusion, our data show the ability of IVIg administration to prevent inflammation and fibrosis in 2 complementary models and a more mitigated effect of a single dose of IVIg on an established model in a curative design. These results open a concrete perspective to consider IVIg as a therapeutic option to be developed in SSc, favoring an early use and more than 1 course. The mechanisms of action remain to be fully deciphered but could include an effect on cytokines and regulation of the immune system. In addition, a further interesting perspective supporting the IVIg administration in the management of SSc patients could be to evaluate also their efficiency in controlling vascular damage, which is a main feature of human disease. For this aim, could be more useful to test the IVIg effects on genetic models, such as the Fos-related antigen-2 (Fra-2) transgenic mouse, which simultaneously display both fibrosis and prominent vascular involvement, differently from the inducible models, such as the HOCl and BLM models, that do not show specific alterations of endothelial components^19^. However, additional studies are mandatory to better assess the mechanisms of action of IVIg in this disease and well-designed randomized control trials are necessary to study the efficacy and tolerance of IVIg in patients with SSc.

## Materials and methods

### Animals

Six-weeks-old female Balb/c AnNRj mice (n = 200) and eight-weeks old female C57BL/6 mice (n = 40) were purchased from Janvier Labs (Le Genest-Saint-Isle, France) and maintained in an accredited pathogen-free facility at the Pasteur Institute of Lille (France) under constant room temperature with a 12-h light/dark illumination and provided with ad libitum access to standard diet and water. Experiments were performed under the European Directive No. 2010/63/EU, revising Council Directive No. 86/609/EEC of November 24, 1986. Ethical approval for this study was obtained from the “Comité d'éthique en expérimentation animale (CEEA 75). NORD PAS-DE-CALAIS, France” with the number #19603–2020061914271271 v6. Animal handling and euthanasia were carried out by qualified personnel for animal experimentation, following the national and institutional guidelines for animal welfare indicated by the French directive No. 2013–118 of 1st February 2013. The experimental designs were conceived and performed in accordance with the ARRIVE guidelines^20^.

### Compounds

The hypochlorous acid (HOCl) solution was extemporaneously generated by adding 9.6% NaClO (Sigma Aldrich, Saint-Quentin-Fallavier, France) to 100 mM KH_2_PO_4_ (pH 6.2) (Sigma Aldrich), in a 1:60 ratio, ensuring a HOCl solution absorbance at 292 nm ranging between 0.7 and 0.9. Bleomycin (BLM) (Sigma Aldrich) was solubilized in 0.9% NaCl at the optimal concentration of 2 mg/Kg.

IVIg (purchased by Octapharma, Lachen, Switzerland) were diluted at the optimal concentration of 2 g/Kg in 2% Maltose, as vehicle.

IVIg, as well as the vehicle, were administered to both control and HOCl or BLM groups.

by a single retro-orbital injection at d0 (with preventive intent) or d21 (with curative intent) of the disease-induction protocol.

### Experimental design

#### Hypochlorous acid (HOCl) experimental model

Skin fibrosis was induced by repeated intradermal injection of 300 µL of HOCl into the shaved back of mice for 5 consecutive days per week, for a cumulative of 3 weeks (d21) or 6 weeks (d42)^21^. 300 µL of PBS were administered by intradermal injection to the control mice, applying the same treatment scheme used in the HOCl groups. Mice were randomized to treatments with PBS + 2% Maltose, PBS + 2 g/Kg IVIg, HOCl + 2% Maltose and HOCl + 2 g/Kg IVIg. The randomization strategy complied with the ARRIVE guidelines and was based on the blocking method, taking into account the cage location as a nuisance variable. The sample size for each group was to ensure adequate statistical power, by considering the previously observed averages of the percentage of disease incidence (70–80%) and the lack of mortality in the HOCl group (Fig. 1a). Thus, 50 Balb/c mice per group were included in this study. For each group (n = 50), 10 mice were treated (IVIg or 2% Maltose) in preventive mode and euthanized at d21, 20 mice were treated (IVIg or 2% Maltose) in preventive mode and euthanized at d42 and 20 mice were treated (IVIg or 2% Maltose) in curative mode and euthanized at d42 (Fig. 1a,b,c). For both preventive and curative mode, skin inflammation and fibrosis were assessed for all mice.

#### Bleomycin (BLM) model

Eight-weeks old C57BL/6 mice were randomized to the following groups: PBS + 2% Maltose (n = 10), PBS + BLM (n = 10), BLM + 2% Maltose (n = 10) and BLM + IVIg (n = 10). Briefly, mice received an intradermal injection of 0.9% NaCl or 2 mg/Kg BLM in 100 µL, into the shaved backs of mice (5 consecutive days per week), for 42 days^22^. As for the HOCl model, IVIg was administered by a single retro-orbital injection with preventive intent (d0). As for the HOCl model, the sample size for each group was to ensure adequate statistical power, by considering the previously observed averages of the percentage of disease incidence (70–80%) and the lack of mortality in the HOCl group (Fig. 1a). For both preventive and curative mode, all mice of each group were used for skin inflammation and fibrosis assessments.

### Clinical parameters: body weight variation and skin thickening

All mice were monitored for body weight variation once per week. Skin thickness was measured using an external caliper, in the shaved back, before the disease induction (d0) and every four days until sacrifice. All measurements, expressed in millimeters, were carried out blindly by a single operator.

### Histological evaluation: May-Grünwald-Giemsa (MGG) and Picrosirius red stainings (PRS)

Four days after the last HOCl (or BLM) injection, all animals from each group were euthanized by cervical dislocation under deep CO_2_ anesthesia. Skin (0.5 cm) was fixed in 4% of buffered formaldehyde solution in PBS at pH 6.9 and paraffin-embedded. After removing paraffin with xylene and rehydration with decreasing serial dilution of ethanol (from 96 to 70%), 4 μm serial sections for each skin sample were incubated for 15 min in May-Grünwald solution and 40 min in Giemsa staining at 37 °C, for microscopic measurements of skin thickness and count of inflammatory cellular infiltrate. Each skin section was also stained using the PRS to evaluate collagen fibers deposition, according to the manufacturer’s protocol. Briefly, 4 μm dried skin sections were stained with 0.1% Direct Red stain (Sigma Aldrich)/0.5% Picric Acid (Sigma Aldrich) for 60 min and mounted after washings in 0.5% Acetic Acid (Sigma Aldrich. Then, serial measurements of the dermal layer, density and extent of immune cells infiltration, as well as collagen fibers production, were performed by acquiring MGG and PRS stained skin sections as digital images on a slide scanner Axioscan Z1 (magnification: × 20). Analysis of dermal thickness, as well as the number of inflammatory cells and the mean red-staining intensity (testifying collagen deposition) were remotely measured by the web-based Image J domain morphometric software (W. S., Rasband, ImageJ, U. S. National Institutes of Health, Bethesda, MD; http://rsb.info.nih.gov/ij/, 1997–2011) by threshold methods associated with color deconvolution plugins. Measurements were performed by two blinded investigators (MJ and MMF).

### Immunofluorescence assay

Detection of alpha-smooth muscle actin (α-SMA)-positive cells was performed on skin sections after 1 h of permeabilization with 0.1% Triton-X 100. Briefly, each section was incubated overnight at 4 °C with a rabbit polyclonal anti-α-SMA primary antibody (ab5694, Abcam Paris, France) at the dilution of 1:200. A specific anti-rabbit green-fluorescent Alexa-Fluor 488 conjugated secondary antibody (Thermo Fisher Scientific) was used 1:10,000. Nuclei were visualized as blue fluorescence by adding 4′,6-diamidino-2-phenylindole dye (DAPI, Thermo Fisher Scientific) to the mounting medium. For three not superimposed microscope fields (magnification: × 20/ × 40) of each section, the percentage of α-SMA-positive cells/total area was performed using the Image J software-based threshold detection method on the grayscale image^23^.

### Quantitative real-time polymerase chain reaction (qRT-PCR)

The gene expression levels of pro-inflammatory cytokines and main markers of fibrosis were assessed in frozen skin samples using quantitative qRT-PCR. Briefly, total RNA was extracted with a Nucleospin RNA kit (Macherey–Nagel, Hoerdt, France), according to the manufacturer’s protocol. Any possible traces of genomic DNA were eliminated via a DNAse treatment then RNA was eluted in RNAse-free, DEPC-free water. For each sample, the purity and amount of the RNA were evaluated by UV spectroscopy on a Nanodrop system from 220 to 350 nm. 1 µg of total RNA was used to perform a qRT-PCR by using LightCycler FastStart DNA Master SYBR Green I from Roche Diagnostics (Indianapolis, IN) according to the manufacturer's protocol. Primer sets included a panel of cytokines including *Il6*, *Il1b* and *Tnf* and main markers of fibrosis, such as *Tgfb*, *Acta2*, *Fn1* and *Col1a1*. The primer sequences are listed in Supplementary Table S1. Relative gene expression was calculated as E = 2^−ΔCt^, with ΔCt being the difference between the critical threshold cycle (Ct) values of each gene and the relative Ct of the reference gene (*Gapdh*). Data were expressed as fold change of the mean relative gene expression values ± SEM between treatment and control groups.

### Hydroxyproline content

Collagen content was assessed by using a colorimetric Hydroxyproline Kit Assay (Sigma-Aldrich) as per the manufacturer's protocol. Briefly, approximately 10 mg of frozen skin samples were homogenized in 100 µl of water and hydrolyzed at 120 °C for 3 h in an equal volume of 12 M hydrochloric acid (HCl). Subsequently, a Chloramine T mixture was added and absorbance was measured at 540–560 nm after 90 min of incubation at 60 °C with 4-(Dimethylamino)benzaldehyde (DMAB). Hydroxyproline assay data (pg hydroxyproline) was normalized to µg of total proteins, determined using a Bradford Assay Kit (Sigma-Aldrich).

### Flow cytometry

Spleen-resident cells were isolated by dissecting and mincing whole spleens from 5 to 10 mice per group. The spleen cell suspension was filtered on 70-µm nylon mesh and erythrocyte depletion was performed using the Hybri-Max, Red Blood Cell Lysing Buffer (Sigma-Aldrich). Then, the number of viable cells was established by flow cytometry using a propidium iodide cell viability assay.

Thus, for each mouse, splenic immune cell subsets (B cells, T cells, NK cells and monocytes/macrophages), as well as B cell subsets were identified by multicolor flow cytometry using specific monoclonal antibodies cocktails. The phenotypic profiling and gating strategy were performed as previously described^14^. Data were then acquired on a 4-laser cytometer (Cytoflex, Beckman Coulter) and analyzed with dedicated software (Kaluza, Beckman Coulter).

### Statistical analysis

All quantitative data were expressed as means ± SEM and analyzed using the GraphPad Prism 8.0.1 software package. For the skin thickness variation (Fig. 1b,d) data were compared using a two-way ANOVA test with Tukey’s multiple comparisons. For the quantification of the histological parameters, the gene expression levels of markers of fibrosis and splenocyte subsets distribution, the one-way ANOVA Kruskal–Wallis test with Dunn’s multiple comparisons was used. The *p* values < 0.05 were considered significant.

### Supplementary Information

Supplementary Figure S1.

Supplementary Table S1.

## Data Availability

Due to confidentiality agreements, the datasets generated and/or analyzed during the current study are not publicly available but are available from the corresponding author on reasonable request.
