# Supplementary material for: Intravenous immunoglobulins improve skin fibrosis in experimental models of systemic sclerosis
Source: Sci Rep. 2023 Sep 12;13:15102. doi: 10.1038/s41598-023-42464-9 (PMC10497569; doi:10.1038/s41598-023-42464-9)
Supplement: Supplementary file 1 — Supplementary Figure S1. [file 41598_2023_42464_MOESM1_ESM.pdf]

Intravenous immunoglobulins improve skin fibrosis in experimental models of systemic sclerosis

Silvia Speca[1], Meryem-Maud Farhat[1,2], Manel Jendoubi[1], Thomas Guerrier[1], Sébastien Sanges[1,2], Delphine Staumont-Sallé[1,3], Eric Hachulla[1,2], Sylvain Dubucquoi[1,4] Vincent Sobanski[1,2], Aurore Collet[1,4], David Launay[1,2,*]

Supplementary Figure S1: Preventive IVIg administration reduces skin fibrosis in BLM-receiving mice

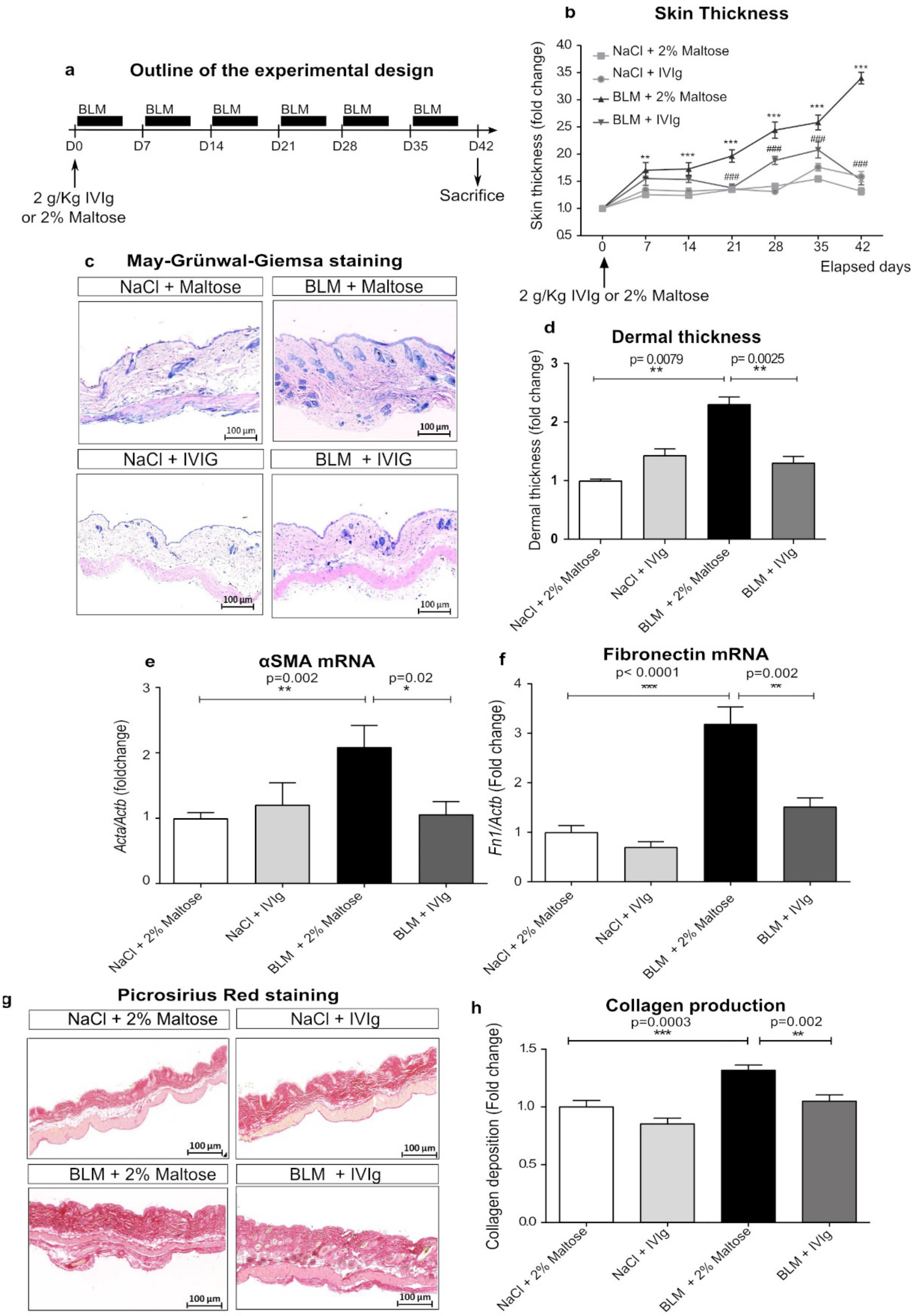

Supplementary Fig S1. Anti-fibrotic effect of preventive IVIg administration in a murine model of BLM-induced SS. (a) Outlines of the experimental design for preventive protocol. Mice were randomized to: PBS + 2% Maltose (n=10), PBS + BLM (n=10), BLM + 2% Maltose (n=10) and BLM + IVIg (n=10). 2 mg/Kg BLM were administered by daily intradermal injection into the shaved backs of mice until sacrifice. 2 g/Kg of IVIg were administered in a single retro-orbital injection at d0 with preventive intent. (b) Curves depicting skin thickness variation during the BLM administration and ata are quoted as mean ± SEM. *= p< 0.05 vs PBS + 2% Maltose; **= p< 0.01 vs PBS + 2% Maltose; ***= p< 0.001 vs PBS + 2% Maltose; ****= p< 0.0001 vs PBS + 2% Maltose; ##= p< 0.01 vs HOCl + 2% Maltose; ###= p< 0.001 vs HOCl + 2% Maltose. Data were analyzed by a Two-way ANOVA test with Tukey's multiple comparisons. (c) Representative microphotographs of MGG stained skin sections scanned at 20X magnification. (d) Histograms depicting Image J software-assisted measures of dermal thickness. Histograms depicting mRNA expression levels of (e) *Acta2* and (f) *Fn1* genes quantified on frozen skin samples by qRT-PCR. (g) Representative microphotographs for Picrosirius Red staining of skin sections for collagen fibers (red) and scanned at 20X magnification. (h) Histograms for collagen deposition quantified on three microscope fields, using the threshold detection method. Data represent the fold change compared to PBS + 2% Maltose group and are quoted as mean ± SEM and analyzed by Kruskal-Wallis test with Dunn's multiple comparisons; *= p< 0.05; **= p< 0.01; ***= p< 0.001; ****= p< 0.0001.
