# Supplementary material for: Intravenous immunoglobulins improve skin fibrosis in experimental models of systemic sclerosis
Source: Sci Rep. 2023 Sep 12;13:15102. doi: 10.1038/s41598-023-42464-9 (PMC10497569; doi:10.1038/s41598-023-42464-9)
Supplement: Supplementary file 2 — Supplementary Table S1. [file 41598_2023_42464_MOESM2_ESM.pdf]

Supplementary Table S1: Mouse Primer sequences for quantitative RT-PCR

**Gene**	**Primer**	**Sequence (5'→3')**
Tgfb1	Forward	CCCGAAGCGGACTACTATGCT
	Reverse	GTTTTCTCATAGATGGCGTTGTTG
Acta2	Forward	CCTGATGGGCAGGTGATC
	Reverse	ATGAAAGATGGCTGGAAGAGAGTCT
Col1a1	Forward	GAGTACTGGATCGACCCTAACCAA
	Reverse	ACACAGGTCTGACCTGTCTCCAT
Fn1	Forward	CGAAGCCGGGAAGAGCAAG
	Reverse	CGTTCCCACTGCTGATTTATCTG
Gapdh	Forward	ATGGGAAGCTTGTCATCAACG
	Reverse	GGCAGTGATGGCATGGACTG
Il1b	Forward	CAACCAACAAGTGATATTCTCCATG
	Reverse	GATCCACACTCTCCAGCTGCA
Tnf	Forward	CCACCACGCTCTTCTGTCTA
	Reverse	GAGGCCATTTGGGAACTTCT
Il6	Forward	GTTCTCTGGGAAATCGTGGA
	Reverse	CAGAATTGCCATTGCACAAC
